# The effect of continuous intravenous norepinephrine infusion on systemic hemodynamics in a telemetrically-monitored mouse model of sepsis

**DOI:** 10.1371/journal.pone.0271667

**Published:** 2022-08-11

**Authors:** Tetsushi Yamashita, Jonathan M. Street, Brianna C. Halasa, Yoshitaka Naito, Takayuki Tsuji, Naoko Tsuji, Naoki Hayase, Peter S. T. Yuen, Robert A. Star

**Affiliations:** Renal Diagnostics and Therapeutics Unit, NIDDK, NIH, Bethesda, Maryland, United States of America; Mahidol University, Faculty of Tropical Medicine, THAILAND

## Abstract

Sepsis, a life-threatening organ dysfunction, results from dysregulated host responses to infection and still has a high incidence and mortality. Although administration of vasopressors to treat septic shock is standard of care, the benefits are not well established. We evaluated the effect of continuous intravenous norepinephrine infusion in a septic cecal ligation and puncture (CLP) mouse model, evaluating systemic hemodynamics and body temperature post-hoc. CLP surgery significantly decreased mean arterial blood pressure (MAP), heart rate, and body temperature within six hours. Continuous norepinephrine infusion (NE+, n = 12) started at the time of CLP surgery significantly increased MAP at 24 and 30 hours and heart rate at 6, 18, 24, and 30 hours after CLP vs CLP alone (NE-, n = 12). However, addition of norepinephrine did not improve survival rate (NE+ n = 34, NE- n = 31). Early (6 hours or earlier, when the animal became visibly sick) MAP did not predict 7-day mortality. However, heart rates at 3 and at 6 hours after CLP/norepinephrine (NE+) were highly predictive of mortality, as also been found in one clinical study. We conclude that limited hemodynamic support can be provided in a mouse sepsis model. We propose that heart rate can be used to stratify severity of illness in rodent preclinical studies of sepsis therapeutics.

## Introduction

Sepsis, defined as life-threatening organ dysfunction resulting from dysregulated host responses to infection [1], still has a high incidence and high mortality. Worldwide data from 2017 estimates 48.9 million incident cases and 11.0 million sepsis-related deaths, representing 19.7% of all global deaths [2]. Despite the study of multiple pathophysiological mechanisms, no effective treatment other than antibiotics and infectious source control is established [3]. Abdominal sepsis is particularly lethal, can persist over 2 weeks, and has a dismal long-term outcome [4]. Because of the patient-to-patient variation in organ damage and complexities of sepsis pathophysiology, and high mortality rate, survival is used as a clinical endpoint for clinical trials, which is reflected in preclinical animal studies. Hypotension is a critical harbinger of sepsic shock. Although administration of vasopressors to treat hypotension is standard of care and included in clinical guidelines [3], the benefits of vasopressors are not universally accepted [5–8]. Thus, decision-making criteria for individual patient are highly individualized, and pressor use varies greatly in practice [5]. A recent randomized controlled trial reported that a high MAP target (80–85 mmHg) had no survival benefit compared with low MAP target (65–70 mmHg) [9]. Thus, the ideal MAP target or whether an even lower MAP target is optimal may not be generalized to the entire population. In the past, dopamine was widely used in hypotensive sepsis because of its alleged organ sparing properties. However, dopamine induced more frequent adverse events than norepinephrine (NE) without additional survival benefit [10]. Thus, norepinephrine is currently the first choice in the clinical guidelines and the most widely used vasopressor.

Mouse models of hypotension are experimentally challenging because both measurement and treatment of hypotension are difficult. Closed loop detection and treatment of hypotension is more feasible in the rat because of the larger body size [11]. In mice, carotid artery catheterization of mice for telemetric blood pressure and heart rate is technically feasible, but difficult requiring much practice; however, intravenous catheterization for infusion of vasopressors in mice is rarely used. In the current study, we performed both procedures in individual mice. We then evaluated the effect of norepinephrine intravenous infusion in a mouse model of antibiotic and fluid treated septic shock (CLP, cecal ligation and puncture) while monitoring the impact of vasopressor treatment on systemic hemodynamics and body temperature.

## Methods

Eight-week old male CD-1 mice (for telemetry study: NE+ n = 12, NE- n = 12, for survival study NE+ n = 34, NE- n = 31), strain 022, were purchased from Charles River Laboratories (Wilmington, MA) and housed in a NIDDK animal facility. We followed National Institutes of Health Guidelines for the Use and Treatment of Laboratory Animals and the NIDDK Animal Care and Use Committee approved all procedures, including high-mortality survival studies (K100-KDB). All animals had free access to water and chow. All anesthesia was performed with isoflurane. All euthanasia was performed under anesthesia, via exsanguination. All researchers had standard training provided by NIH, as well as individualized, specialized training for these studies.

### Telemetry implantation and catheter insertion

**Fig 1:**
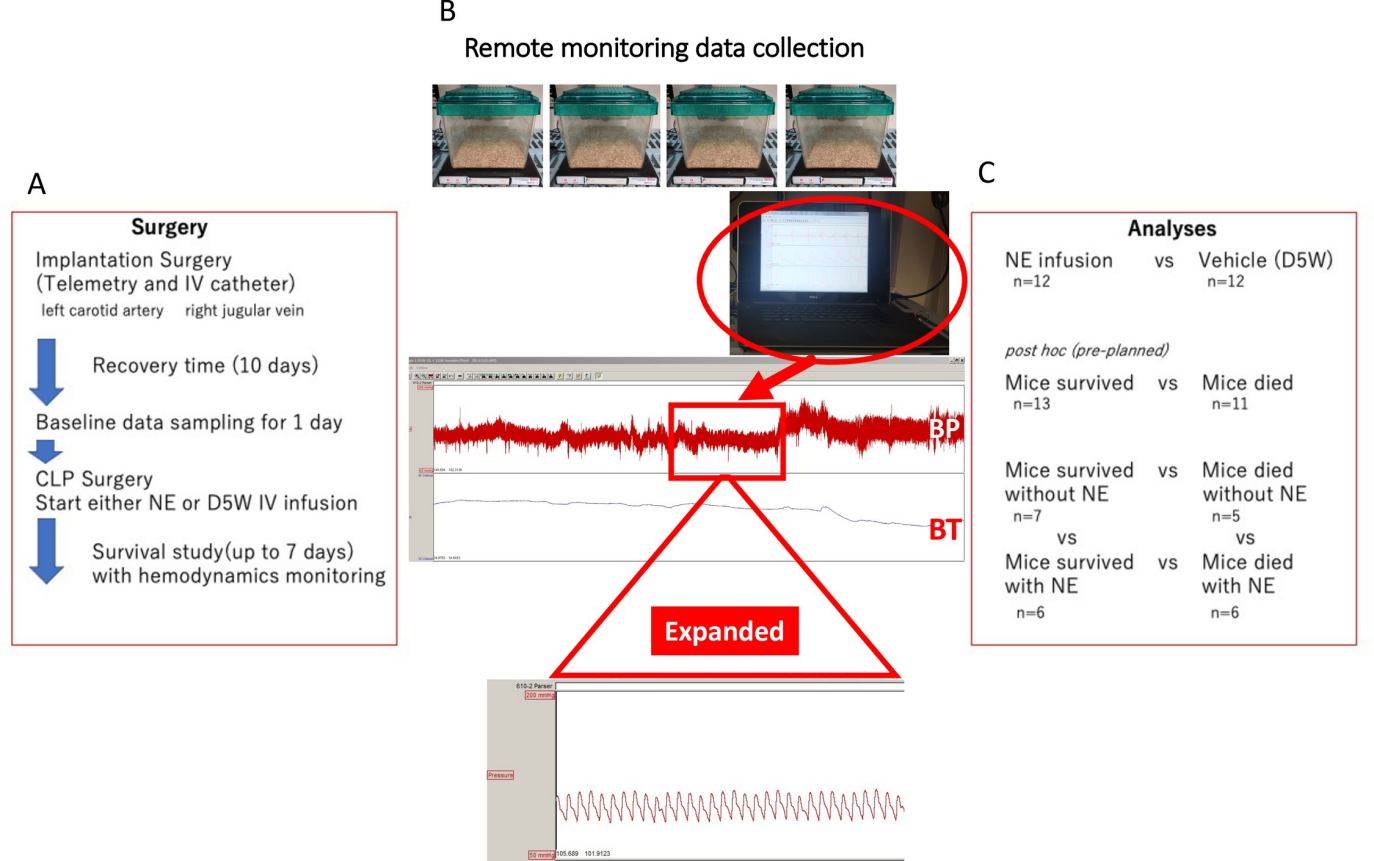
Flow chart and analyses design. IV: intravenous, CLP: cecal ligation puncture, NE: norepinephrine, D5W: 5% dextrose in water, BP: blood pressure, BT: body temperature.

Blood pressure, heart rate, and body temperature were measured with a radio-telemetry probe (HD-X10; Data Sciences International, St. Paul, MN). Briefly, under isoflurane anesthesia, a neck incision was made, and the tip of the telemetry catheter was inserted into the left carotid artery and secured by ligation with 6–0 silk suture as previously described with minor modifications [12, 13]. A second catheter was inserted into the right jugular vein [14] for administration of vehicle (5% dextrose in water: D5W, NE-) or norepinephrine (NE+), and connected to an osmotic minipump initially filled with D5W. The transducer/telemetry transmitter and the osmotic minipump were placed subcutaneously in the back (Fig 1).

### CLP and norepinephrine administration

We followed the recommendations of the recent minimum quality threshold in pre-clinical sepsis studies (MQTiPSS) [15]. Ten days after telemetry implantation, cecal ligation and puncture was performed to induce sepsis, as previously described with minor modifications [16]. Briefly, under isoflurane anesthesia a midline incision was made, and the cecum was located and removed from the peritoneal cavity. The cecal contents were palpated to the tip and the distal 15 mm, filled with contents, were ligated with 4–0 silk suture. A 20 g needle was then passed through the ligated cecum allowing the contents to leak out through two holes. The cecum was returned to the peritoneal cavity and the incision closed. Mice whose body weight decreased over 5 g compared with the body weight at the implantation surgery were excluded (None of the mice fulfilled this exclusion criteria). Mice were assigned based on matching body weights in each group to either with norepinephrine administration (NE+) after CLP surgery (*n* =  12) or without norepinephrine administration (NE-) after CLP surgery (*n* =  12). The order of mice was mixed between groups and cages (4 mice/cage). The osmotic minipump implanted subcutaneously was replaced with another osmotic minipump prefilled with either norepinephrine (NE+) or vehicle (D5W: NE-). The norepinephrine concentration was adjusted so that the dosage was 3 ug/kg/min. Initial studies with 10 and 35 ug/kg/min showed increased variability or hypertension/mortality, respectively (S1 Fig). Prior to implantation the dead space of the jugular vein catheter was flushed with norepinephrine solution in the group with norepinephrine (NE+) so that norepinephrine administration could start soon after CLP (Fig 1). All mice received a slow-release formulation of buprenorphine (SR Veterinary Technologies, Windsor, CO) subcutaneously for analgesia during CLP surgery and every 72 hours thereafter. Fluids (40 mL/kg) were given intraperitoneally at the time of surgery (0.9% saline) and then subcutaneously at 6 hours, and every 12 hours thereafter (0.6% saline). Antibiotics were given with fluids starting at 6 hours (14 mg/kg Primaxin [imipenem and cilastatin, Merck & Co, Whitehouse Station, NJ] and then 7 mg/kg every 12 hours).

### Survival study, monitoring, and euthanasia

For the survival study, mice without telemetry were included for analysis. Sixty-five mice were used; 34 were with treated with norepinephrine (NE+) and 31 were not treated with norepinephrine (NE-). These sample sizes were calculated to detect a 30% or greater absolute difference in survival between CLP groups with 80% power and α  =  0.05 [17]. All mice were monitored at 6, 18, and 24 hours, and from 24–72 hours, at least every 4 hours during the day and at least every 6 hours at night, and then every 8 hours until 7 days, when the study was concluded. Mice were given a euthanasia score based on their respiratory pattern (depressed respiratory rate: 2, or apneustic respiration: 5), activity (spontaneous activity without stimulus: 0, activity in response to tactile stimuli: 1, delayed activity in response to tactile stimuli: 2, or unresponsive to tactile stimuli: 5) and general appearance (piloerection: 1, lack of eye grooming: 1) Animals with a score > 5 are humanely euthanized (8). Clinical activity score was scored separately, from 0 (no activity after stimuli) to 6 (active as normal) according to mice activity [18] based on a more extensive scoring system [19]. The surgeon, who also monitored mice after surgery, was aware of which groups mice belonged to (no blinding). Euthanasia was performed under isoflurane anesthesia, where blood was collected, and lack of heartbeat was confirmed. In this study, none of mice met the criteria for euthanasia. Thirty-six mice died within 7 days; 20 of them were treated with norepinephrine (NE+) and 16 of them were not treated with norepinephrine (NE-). Of the catheterized mice (n = 24) eleven died during the 7 day duration of the experiment, and they died within three hours of the previous monitoring, with a euthanasia score below 5.

### Statistics and data analysis

Continuous blood pressure, heart rate, and body temperature data was averaged across each minute and transmitted telemetrically (Fig 1). To reduce variability, we averaged consecutive 10 min time windows. Baseline values were measured at the same time of day (2:00 pm). After CLP, times were synchronized by time after CLP surgery. The normality of data was assessed with the Shapiro-Wilk test (Prism 8.4.1; GraphPad Software, CA). For variables with a normal distribution, t-test, paired t-test, ANOVA, and one-way ANOVA were used; otherwise, the corresponding non-parametric tests were used. The prediction ability of data for mortality was assessed using receiver operating characteristic (ROC) curve analysis. The null hypothesis was rejected if *P* < .05.

## Results

Sepsis was studied in young outbred CD-1 mice with post-hoc analysis of hemodynamics and body temperature. As mean arterial blood pressure, heart rate, and body temperature are sensitive to circadian variation [20, 21], baseline values were obtained at the same time of day. The norepinephrine concentration was adjusted so that the dosage was 3 ug/kg/min. In initial studies using doses of 10 and 35 ug/kg/min, we found increased variability or hypertension/mortality, respectively (S1 Fig). Because the first mouse died approximately 39 hours after CLP, we analyzed the hemodynamic data from baseline to 36 hours after CLP.

### Effect of CLP on hemodynamic parameters

**Fig 2:**
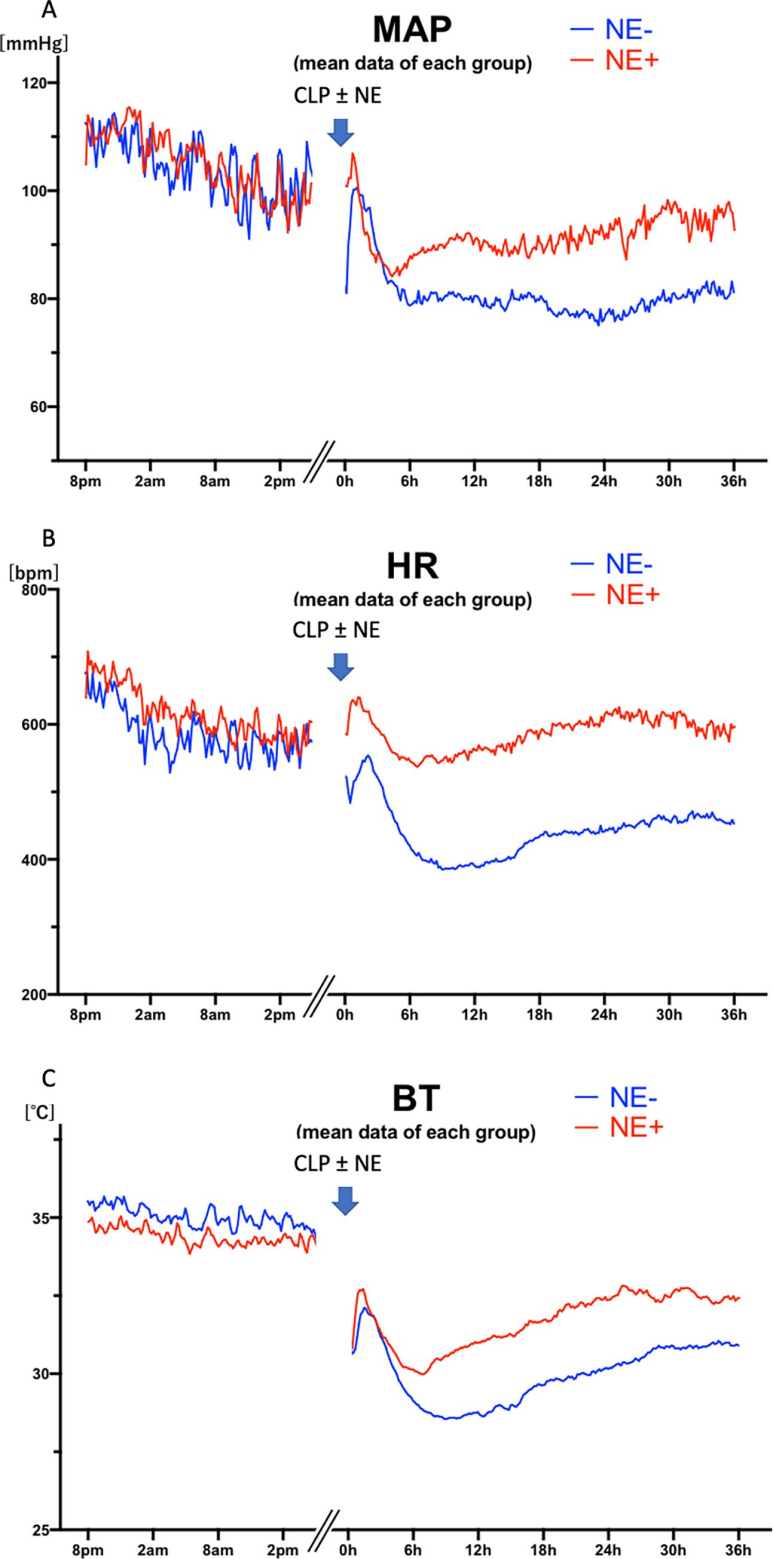
Each line shows the mean data of each group until 36 hours after cecal ligation puncture (CLP): Vehicle-treated mice (blue; n = 12), and mice treated with norepinephrine (NE) (red; n = 12). A: mean arterial blood pressure (MAP), B: heart rate (HR), C: body temperature (BT) baseline values are synchronized by time of day; after CLP, times are synchronized by time after CLP surgery. A gap between baseline data collection and post-CLP/infusion is indicated.

**Fig 3:**
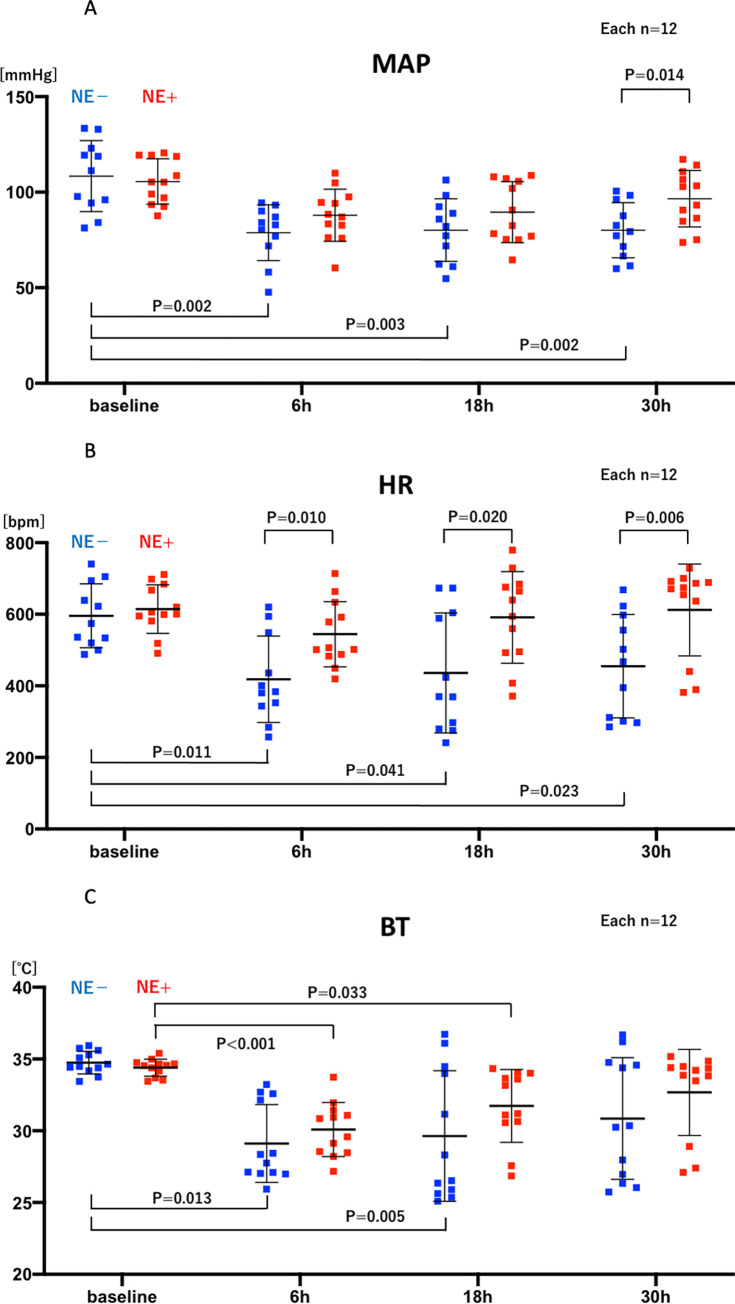
The group with norepinephrine (NE+) treatment (red; n = 12) is compared with the group without NE (NE-)treatment (blue; n = 12) at baseline (2:00 pm), and 6, 18, 30 hours after CLP to evaluate NE effects on MAP, HR, and BT. All significant differences are shown with their p value. A: mean arterial blood pressure (MAP), B: heart rate (HR), C: body temperature (BT). t-test, Mann-Whitney’s U test, one-way repeated measures ANOVA, and Friedman test were used.

The average continuous hemodynamic data (Fig 2) and averages at specific times (Fig 3) of mean arterial blood pressure (MAP, Figs 2A and 3A), heart rate (HR, Figs 2B and 3B), and subcutaneous body temperature (BT, Figs 2C and 3C) are shown for control (vehicle, NE-) and norepinephrine treated (NE+) mice. After increases post-anesthesia, perhaps reflecting the warm shock phase, all parameters decreased substantially within six hours after CLP. As expected, CLP without vasopressor support (NE-) significantly decreased MAP, HR, and BT at 6 hours (108±19 baseline vs 79±15 mmHg CLP, p = 0.021; 596±89 vs 418±121 bpm p = 0.011; 34.7±0.8 vs 29.1±2.7°C, p<0.001, n = 12) (Fig 3). The bradycardia and hypothermia are typically found in mouse sepsis models [12, 22–27], although not a part of human sepsis, except in severe and terminal phases [28, 29].

### Effect of norepinephrine on hemodynamic parameters in CLP treated mice

NE significantly increased MAP at 24 hours (77±13 vs 93±21 mmHg, p = 0.042, n = 12 each) and 30 hours (80±14 vs 97±15 mmHg, p = 0.014, n = 12 each) and HR at 6 hours (418±121 vs 544±91 bpm p = 0.010, n = 12 each), 18 hours (436±168 vs 591±128 bpm p = 0.020), 24 hours (443±141 vs 617±127 bpm p = 0.005, n = 12 each), and 30 hours (455±144 vs 612±128 bpm p = 0.006, n = 12 each) after CLP, whereas even with norepinephrine (NE+) treatment, CLP significantly decreased BT at 6 hours (34.4±0.6 vs 30.1±1.9°C, p<0.001, n = 12), 18 hours (34.4±0.6 vs 31.7±2.5°C, p = 0.033, n = 12), and 24 hours (34.4±0.6 vs 32.3±2.5°C, p = 0.033, n = 12), compared with baseline.

### Effect of norepinephrine on mortality

**Fig 4:**
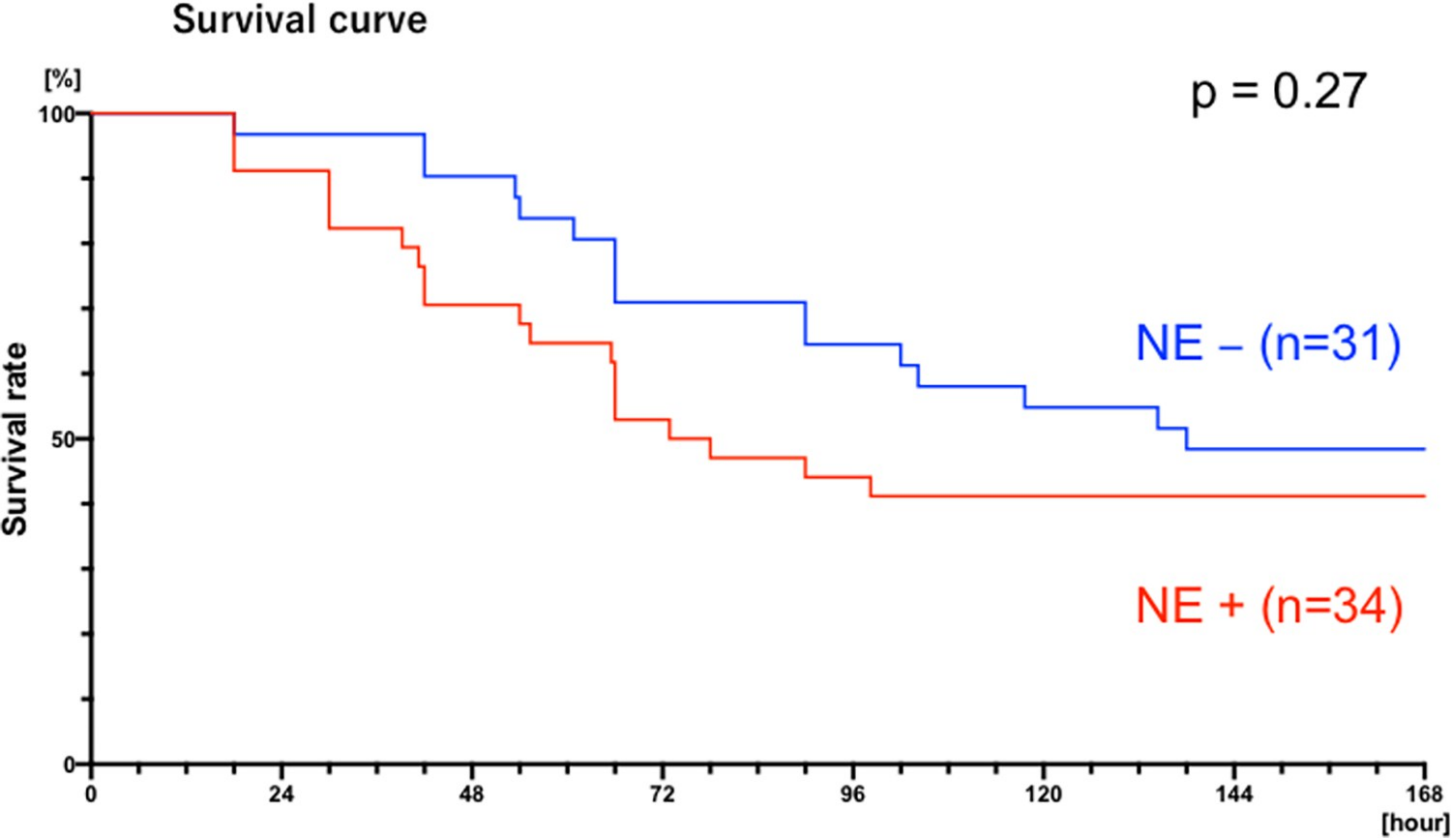
Kaplan-Meier survival curve of norepinephrine (NE)-treated mice (red; n = 34) compared with vehicle-treated mice (blue; n = 31).

Approximately half of the mice died within 7 days after CLP; 58.8% (42.2–73.7%, 95% CI) with norepinephrine (NE+) and 51.6% (34.8–68.0%, 95% CI) without norepinephrine (NE-). In a Kaplan-Meier survival analysis, the two curves were not significantly different (p = 0.27, NE+ n = 34, NE- n = 31) (Fig 4). The timing of mortality appeared comparable between the two groups, which allowed us to compare the predictive value of early MAP, HR, and BT on time of death (see below).

### The difference between survivors and mice that died within 7 days, independent of NE treatment

**Fig 5:**
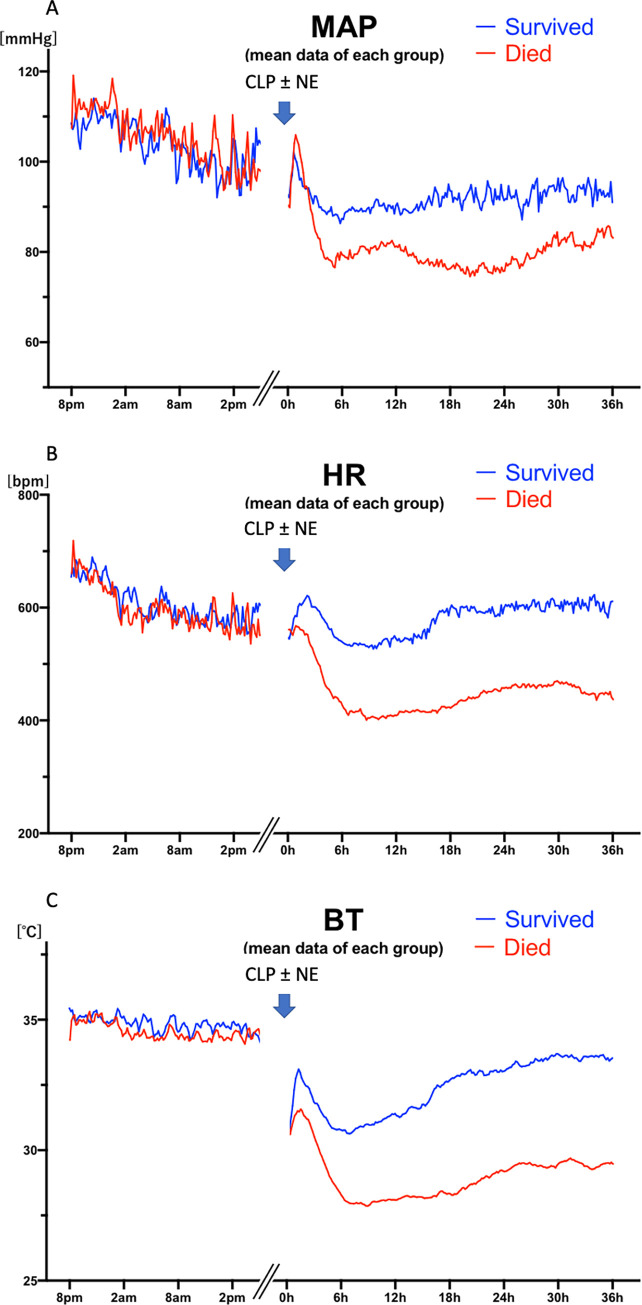
Each line shows the mean data of each group from baseline until 36 hours after cecal ligation puncture (CLP), including survivors (blue; n = 13), and mice that died within 7 days (red; n = 11) regardless of norepinephrine (NE) administration. A: mean arterial blood pressure (MAP), B: heart rate (HR), C: body temperature (BT) baseline values are synchronized by time of day; after CLP, times are synchronized by time after CLP surgery.

**Fig 6:**
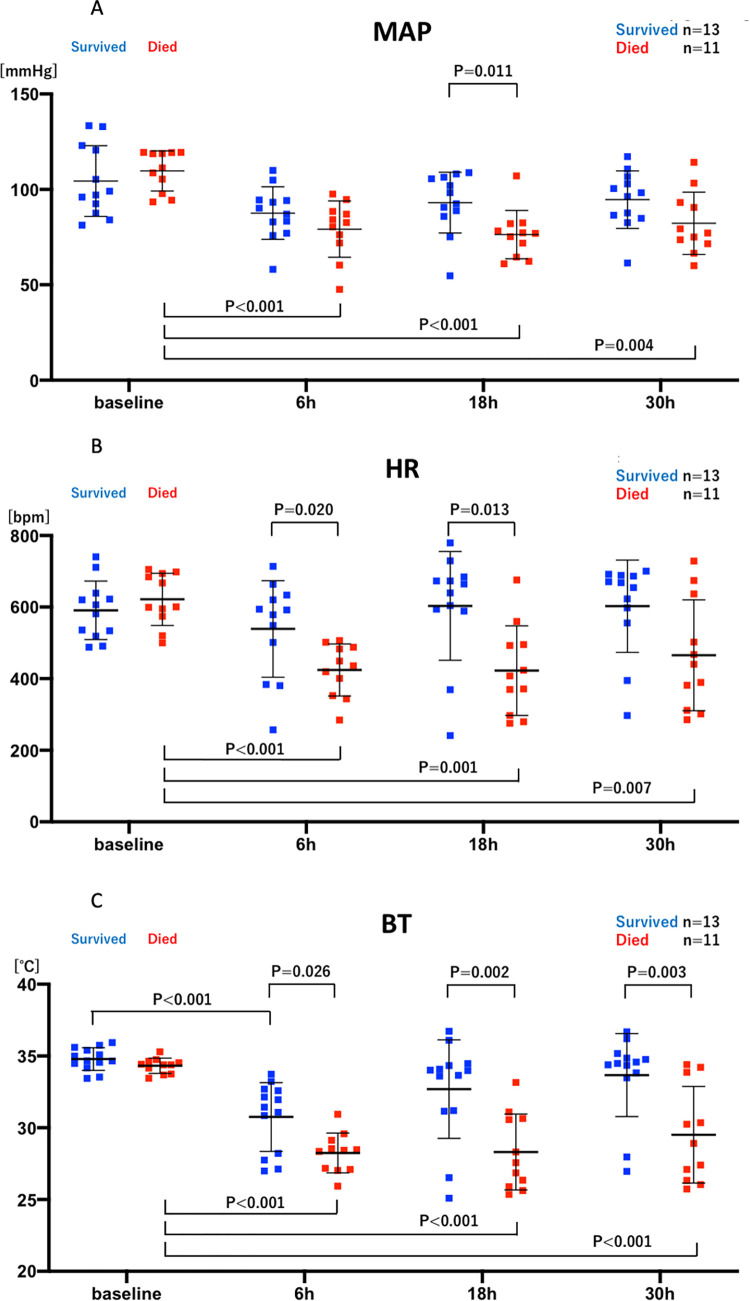
Survivors (blue; n = 13) are compared with mice that died within 7 days (red; n = 11) regardless of norepinephrine (NE) administration, at baseline, and 6, 18, 30 hours after cecal ligation puncture (CLP). All significant differences are shown with their p values. A: mean arterial blood pressure (MAP), B: heart rate (HR), C: body temperature (BT). t-test, Mann-Whitney’s U test, one-way repeated measures ANOVA, and Friedman test were used.

We noticed large differences in HR and BT between mice that survived and mice that died. Survivors had significantly higher HR (at 6 and 18 hours, Figs 5B and 6B, n = 13 survived vs n = 11 died), and BT (at 6, 18, and 30 hours, Figs 5C and 6C, n = 13 survived vs n = 11 died) than mice that died within 7 days. Although there were consistent trends where survivors had higher MAP (Fig 5A, n = 13 survived vs n = 11 died), significant differences were detected only at 18 hours (Fig 6A, n = 13 survived vs n = 11 died).

### Combined analysis of NE treatment x survival within 7 days

**Fig 7:**
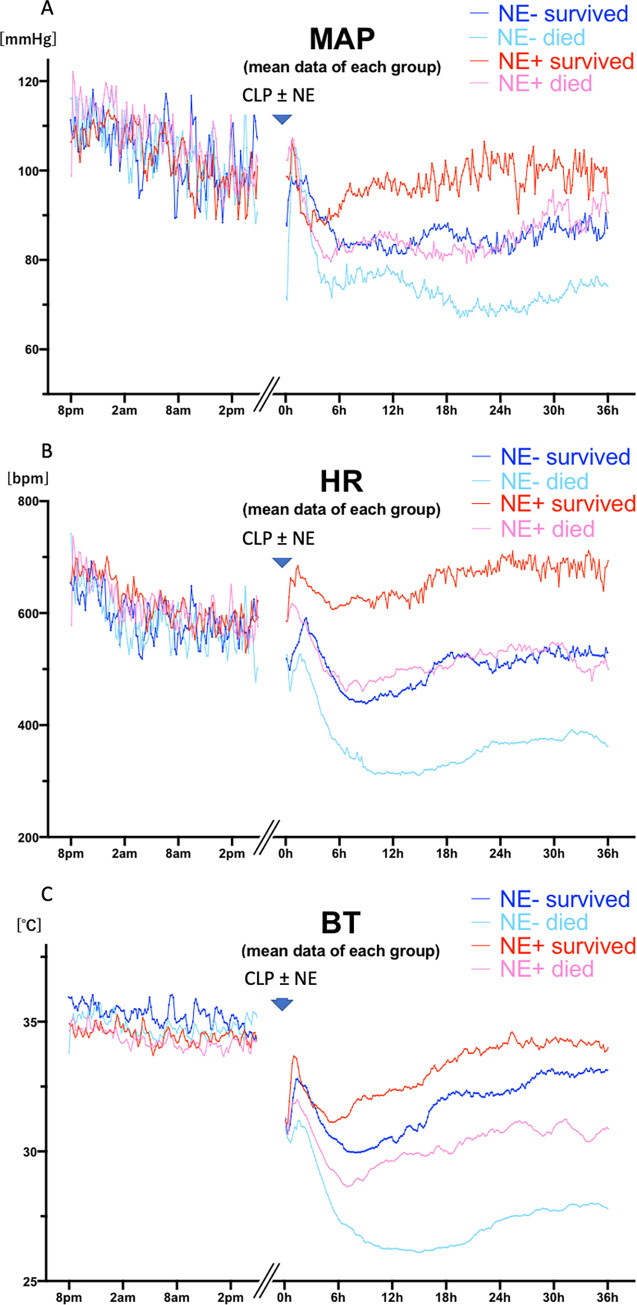
Each line shows the mean data of each group until 36 hours after cecal ligation puncture (CLP): Survivors without norepinephrine (NE) treatment (dark blue; n = 7), mice that died within 7 days, without NE treatment (light blue; n = 5), survivors with NE treatment (dark red; n = 6), and mice that died within 7 days, with NE treatment (light red; n = 6). A: mean arterial blood pressure (MAP), B: heart rate (HR), C: body temperature (BT) baseline values are synchronized by time of day; after CLP, times are synchronized by time after CLP surgery.

**Fig 8:**
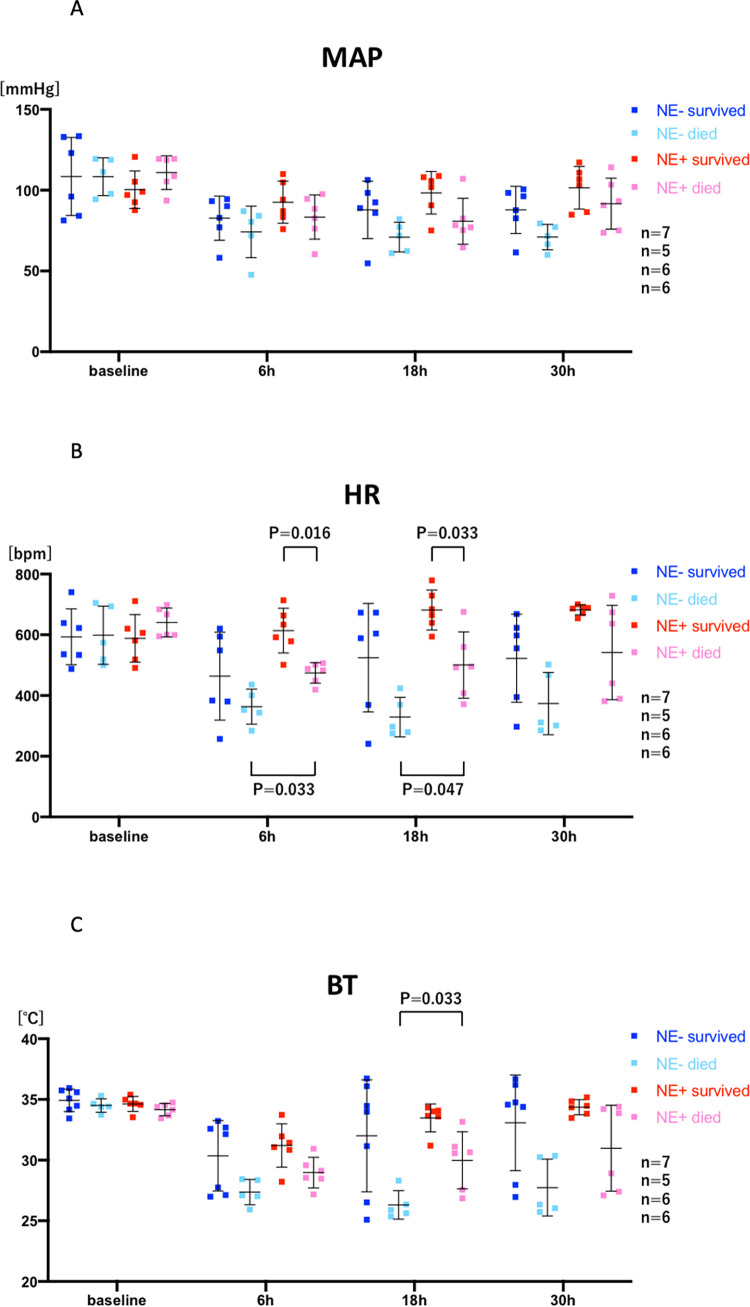
Comparison of 4 groups: Survivors without norepinephrine (NE) treatment (dark blue; n = 7), mice that died within 7 days, without NE treatment (light blue; n = 5), survivors with NE treatment (dark red; n = 6), and mice that died within 7 days, with NE treatment (light red; n = 6) at baseline, and 6, 18, 30 hours after cecal ligation puncture (CLP) are shown. All significant differences are shown with their p values. A: mean arterial blood pressure (MAP), B: heart rate (HR), C: body temperature (BT). ANOVA, Kruskal-Wallis test and Tukey’s post hoc test were used.

Due to the large individual variation in response to CLP and norepinephrine, we divided mice into 4 groups; 1) mice that survived for 7 days without NE treatment (NE-) (n = 7), 2) mice that died within 7 days without NE treatment (NE-) (n = 5), 3) mice that survived for 7 days with NE treatment (NE+) (n = 6), and 4) mice that died within 7 days with NE treatment (NE+) (n = 6). Although there was a consistent trend where survivors had higher MAP, HR, and BT than mice that died within 7 days, and mice with NE treatment (NE+) had higher values than mice without NE treatment (NE-), differences were not statistically significant (Fig 7). Indeed, individual mice with very low MAP, HR, and BT survived without NE treatment (NE-), and some NE-treated (NE+) mice with high hemodynamic values died. However, heart rate (HR) did allow some distinctions. For example, the HR of mice that died within 7 days with NE treatment (NE+) was significantly lower than survivors with NE treatment (NE+) and higher than mice that died within 7 days without NE treatment (NE-) at 6 and 18 hours (Fig 8B). In contrast, the HR of mice that died within 7 days without NE treatment (NE-) could not be distinguished from HR of survivors that were treated with NE (NE+) at 6, 18, or 30 hours (Fig 8B). Therefore, further subdivision into four groups did not substantially improve predictive ability of survival, with (NE+) or without NE (NE-) treatment, although HR outperformed BT and MAP (Fig 8A and 8C).

### The correlation between clinical activity score and hemodynamic factors

Mice were monitored at 6, 18, and 30 hours post-CLP and assigned a clinical activity score from 0 (no activity even after stimuli) to 6 (active without stimuli, as normal mice). We analyzed the correlation among clinical activity score, MAP, HR, and BT. Every pair was significantly correlated (S2 Fig). The correlation between HR and BT were surprisingly strong at all time points, whereas MAP was less correlated with HR or BT. This closer association is consistent with HR and BT being biomarkers for the severity of sepsis in this mouse model; whereas MAP during the early stage of CLP seems to be influenced by factors other than severity of disease (as reflected in the clinical score). As recovery time increased, clinical score became more strongly correlated with each hemodynamic variable.

### Correlative prediction of time of death at 3, 6, 18, and 30 hours after CLP

Given the strong correlation between hemodynamic variables, we searched for predictors of time of death, particularly at early time points (3 or 6 hours). S3 Fig. shows the association between clinical scores or hemodynamic data at 3, 6, 18, and 30 hours after CLP and time of death. In vehicle-treated (NE-) CLP mice, receiver operator characteristic (ROC) curve analysis showed that clinical score and hemodynamic parameters (especially HR) were limited in the ability to predict death. In contrast, in CLP mice treated with NE (NE+), HR in the early stage of CLP consistently had a high predictive value for predicting death, followed by BT and clinical score (S1 Table). Although NE did not increase survival, NE improved the predictive value for mortality of HR and BT in addition to clinical score. The predictive value with NE treatment (NE+) was improved when the clinical score and hemodynamic parameters were used in combination (S2 and S3 Tables). The earliest and strongest predictor that we found was the HR achieved 3 hours after norepinephrine treatment (NE+) (Fig 7B), which predicted mortality with high AUROC value above 0.90 even within 6 hours after CLP (S1 Table).

## Discussion

Clinical guidelines recommend norepinephrine as the first choice to treat hypotension in septic patients [3], yet in most mouse models of sepsis blood pressure is not measured and hypotension is not treated. Our previous models of murine polymicrobial sepsis employed fluid resuscitation and antibiotics [17], and had severe hypotension [30]. In this current study we added a simultaneous continuous intravenous infusion of norepinephrine. We found that norepinephrine infusion increased heart rate, and eventually blood pressure; however, we did not detect a change in mortality (around 50% in both groups). Clinical assessments and hemodynamic assessments were correlated. In the absence of norepinephrine, clinical and hemodynamic measurements were marginally able to predict time of death. However, an increased response of heart rate and body temperature to norepinephrine treatment, as early as three hours post-treatment, was able to predict improved survival. Heart rate, and to a lesser extent body temperature, in response to norepinephrine, clearly outperformed blood pressure as a predictor of survival. There is a close association between bradycardia and hypothermia in the mouse CLP model [23]. Norepinephrine treatment exacerbates white adipose tissue browning, which can cause thermogenesis, in murine CLP [31]. The effect of NE on body temperature, and subsequently heart rate, could partly result from NE-induced white adipose tissue browning. Although it was originally planned to investigate the association between hemodynamic parameters and mortality, these were post-hoc analyses. The results should be considered exploratory and hypothesis-generating.

### Model

Devising mouse models that mimic human sepsis has been quite challenging. Mice are resistant to endotoxin, and thus many in the field have migrated to cecal ligation and puncture models that mimic an abdominal injury with polymicrobial infection. Our experimental design was chosen to mimic the ICU treatment of human septic shock as closely as possible, where fluids, antibiotics, and intravenous vasopressor infusions are standard of care. Many, but not all publications, employ both fluid resuscitation and antibiotics. However, the documented severe hypotension that occurs following CLP is rarely measured, and is always untreated. In the current study we added a simultaneous continuous intravenous infusion of norepinephrine. We chose to use norepinephrine because guidelines recommend norepinephrine as the first choice to treat hypotension in septic patients [3] and an observational study indicated norepinephrine benefit compared with phenylephrine in patients with septic shock [32]. Although we tried to mimic the ICU treatment of human septic shock as closely as possible, there were several differences, including fluid administration with fixed volume and timepoint, fixed dose of norepinephrine, and no other monitoring than clinical appearance, BP, HR, and BT.

### Effect of norepinephrine on heart rate

Unexpectedly, NE had a substantially greater effect to improve sepsis-induced bradycardia compared to sepsis-induced hypotension in mice. This might be related to the CLP-induced bradycardia in mice, in contrast to the usual tachycardia during human sepsis in response to bacteremia, fever, or vasodilation. Recently, the heterogeneity of sepsis has attracted attention as a potential reason why most randomized controlled trials of intervention have not identified a treatment benefit [33, 34]. Several subphenotypes of sepsis have been reported [25, 35–39]. Among subphenotypes based on body temperature trajectories, hypothermic patients with septic shock had the highest mortality, ~50% [38, 39]. Our mouse model with high mortality (~50%) likely falls into the hypothermic subphenotype. Among subphenotypes based on cardiovascular parameters, the “well resuscitated” group had the highest heart rate after initial fluid resuscitation and vasopressor infusion, and the best prognosis [34]. Norepinephrine simulates sympathetic activity, not only as an α-adrenergic agonist but also as a β-adrenergic agonist [40, 41]. In clinical studies in septic shock, norepinephrine caused a significantly higher heart rate [42] and more arrhythmias [43] than vasopressin, and improved cardiac systolic function despite the presumed increase in left ventricular afterload [44]. In human sepsis, NE dose is carefully titrated in real time to mean blood pressure targets [3, 9], and this minimizes potential harm [9]. However, our mouse data suggests that achieving a target heart rate in response to NE may be a better marker of the overall trajectory of sepsis.

Why should heart rate outperform blood pressure to predict mortality? Blood pressure may be determined by multiple factors (e.*g*., pre-load, afterload, cardiac output), whereas HR may have fewer determinants or can be overridden by β2-adrenergic receptor input. Considering that heart rate had surprisingly high predictive value for mortality, even in the early stage with norepinephrine treatment, we propose a closer examination of heart rate in patients during the early phase of sepsis, especially when norepinephrine dose is adjusted, in order to predict mortality. Our hypothesis still needs to be corroborated by future clinical studies that determine the association between the initial heart rate response to norepinephrine with the prognosis of septic patients.

### Lack of survival benefit

Continuous intravenous norepinephrine administration did not improve survival rate in CLP, although it significantly increased blood pressure and heart rate during sepsis. In sheep, norepinephrine alone did not ameliorate increases in lactate and organ injury, whereas vasopressin alone or vasopressin with norepinephrine did. Every treatment, to a lesser extent norepinephrine alone, increased survival time than no vasopressor [45]. A RCT also failed to show early norepinephrine treatment improved survival rate compared with standard treatment, although it had significant higher shock control rate at 6 hours after randomization [46]. There are a number of potential explanations for why NE might not have a net survival benefit.

Previous studies reported norepinephrine can have immunosuppressive effect via β2-adrenergic receptor against lipopolysaccharide (LPS) stimulation in mice [47, 48] and humans [48, 49]. One of these studies [48] also showed norepinephrine infusion increased bacterial dissemination in the liver, spleen, and blood in mice subjected to CLP. This would be consistent with the tendency of earlier deaths of mice with NE vs without NE treatment in the present study. In contrast, catecholaminergic vasopressors like epinephrine and norepinephrine reduce LPS-induced microvascular endothelial cell permeability via β-adrenergic receptors, whereas vasopressin or angiotensin II did not reduce permeability [50]. β2-adrenergic receptor is also part of the cholinergic anti-inflammatory pathway which has a protective effect against AKI in a kidney ischemia-reperfusion injury (IRI) model [51]. These complex manifestations cannot be readily addressed in our model system. Rather, our model is better suited to test and compare other pressors, including supplementing norepinephrine with other pressors, as is done currently in clinical trials, comparing standard of care vs standard of care plus another pressor. Vasopressin, a non-catecholamine vasopressor, is decreased in some septic patients [52, 53] and had been evaluated as an additional vasopressor for refractory hypotension to reduce catecholamine doses that might cause organ ischemia. However, adding vasopressin is not more efficacious than increasing norepinephrine dosage, except for a selected subgroup of patients receiving <15 ug/min norepinephrine at randomization [42], nor is vasopressin monotherapy as the initial vasopressor more useful than norepinephrine monotherapy [54]. Angiotensin II is another promising non-catecholamine direct acting vasopressor that significantly increased blood pressure in patients with refractory hypotension [55], and improved survival in patients with acute kidney injury (AKI) requiring renal replacement therapy [56]. Although our model is labor-intensive, we suggest that the most promising sepsis therapies be tested under the conditions of this pressor-treated and monitored model, so as not to develop a candidate that no longer has benefit when tested in a pressor-treated mouse model.

### Limitations

This study has several limitations. First, this was a study using mice, not a clinical study. The mechanism of norepinephrine may be different in mice and humans. Indeed, HR was not a better predictor of mortality in canine [57] and ovine [58] sepsis models. Differences between large and small animal models can be used to evaluate how norepinephrine and other vasopressors work. Another study conducted using a different telemetry system, notably with core body temperature, in C57BL/6 mice required a combination of both heart rate and temperature to predict mortality [23]. Therefore, there may be differences among mice strains; several effects, including pain sensitivity [59], sepsis severity [60], and baseline blood pressure [21] are strain-dependent. Secondly, norepinephrine dosage might not be optimal, as we cannot adjust the timing or dose as is done routinely as a standard of care [3, 9]. Because we had to set the dose when the minipump was implanted, well before the onset of sepsis. Thus, norepinephrine dosage could not be adjusted to sepsis severity, and is further affected by mice body temperature because drug release by osmotic minipump is temperature dependent (Q = Q_0_[0.135e^(0.054T)^-0.004π+0.03], Q: the actual pumping rate, Q_0_: the specified pumping rate at 37°C, T: ambient temperature, π: the osmotic activity of the pump environment). A drop in BT from 35°C to 26°C would therefore result in a 38% decrease in drug release rate. The dosage used, 3 ug/kg/min, is relatively high compared with dosages used in humans. However, a previous study reported the plasma norepinephrine levels with this dosage is comparable to those in patients with septic shock and trauma treated with norepinephrine [48]. Fluid resuscitation was performed subcutaneously (which is standard of care in murine models), but intravenous fluid resuscitation titrated to the severity/conditions might have been more effective and closer to standards of care for patients. Finally, we only used a small number of animals because of the technical difficulty of the experiments. Our study is underpowered to detect less than a 30% difference in absolute survival rates with norepinephrine treatment. Although our study was originally planned to investigate the association between hemodynamic parameters and mortality, these were post-hoc analyses. Both false positive and negative results could happen. The results should be considered exploratory and hypothesis-generating.

## Conclusion

We established a pressor-treated model of sepsis in mice by simultaneously implanting a telemetry catheter into the carotid artery and an infusion pump into the jugular vein. By post-hoc analysis CLP decreased blood pressure, heart rate, and body temperature corresponding to the severity of sepsis. Continuous intravenous norepinephrine administration significantly increased blood pressure and heart rate during sepsis, but it did not improve survival rate in CLP. With norepinephrine treatment, heart rate at 3 or 6 hours after CLP had high predictive value for mortality, which clearly outperforms the increase in blood pressure for predictive value. While this study has a number of limitations, we propose that the heart rate increase after norepinephrine can be used to stratify severity of illness in preclinical rodent studies for sepsis therapeutics. As our main findings are based on a post-hoc analysis, a prospective study powered for the suggested endpoint "heart-rate as a predictor for mortality" should be performed.

## Supporting information

S1 Fig Preliminary dose finding study for norepinephrine infusion. Different doses of norepinephrine were tested. Each line shows the data of each mice until 24 hours after cecal ligation puncture (CLP): mice treated with 35 ug/kg/min norepinephrine (NE) (red, light red, orange and purple; n = 4), and mice treated with 10 ug/kg/min NE (blue, light blue and pale blue; n = 3). A: mean arterial blood pressure (mean BP), B: heart rate (HR), times are synchronized by time after CLP surgery.
(TIF)

S2 Fig Cross-correlations of clinical score, MAP, HR, and BT at 6, 18, and 30 hours after cecal ligation puncture (CLP). Each color indicates groups: survivors without norepinephrine (NE) treatment (dark blue; n = 7), mice that died within 7 days, without NE treatment (light blue; n = 5), survivors with NE treatment (dark red; n = 6), and mice that died within 7 days, with NE treatment (light red; n = 6). Clinical score ranged from 0 (no activity after stimuli) to 6 (active as normal). CS: clinical score, MAP: mean arterial blood pressure, HR: heart rate, BT: body temperature. Spearman’s correlation is used.
(TIF)

S3 Fig Correlative prediction of time of death at 3, 6, 18, and 30 hours after cecal ligation puncture (CLP). Each color indicates groups: survivors without norepinephrine (NE) treatment (dark blue; n = 7), mice that died within 7 days, without NE treatment (light blue; n = 5), survivors with NE treatment (dark red; n = 6), and mice that died within 7 days, with NE treatment (light red; n = 6). Clinical score was scored from 0 (no activity after stimuli) to 6 (active as normal). CS: clinical score, MAP: mean arterial blood pressure, HR: heart rate, BT: body temperature.
(TIF)

S1 Table AUROC of each parameter/time point for prediction of mortality. (TIF)

S2 Table AUROC of each pair of parameters/time point for prediction of mortality. To weight each parameter approximately equally, we used CS divided by 6, MAP divided by 150, HR divided by 800, and BT divided by 38 for analysis.
(TIF)

S3 Table AUROC of combination of more than three parameters/time point for prediction of mortality. To weight each parameter approximately equally, we used CS divided by 6, MAP divided by 150, HR divided by 800, and BT divided by 38 for analysis.
(TIF)

S1 File ARRIVE guidelines. (PDF)

S2 File Primary telemetry data. Data were collected from telemetric devices for blood pressure, heart rate, and body temperature at 1 min intervals.
(XLSX)
