# Supplementary figures and images for: The effect of continuous intravenous norepinephrine infusion on systemic hemodynamics in a telemetrically-monitored mouse model of sepsis
Source: PLoS One. 2022 Aug 11;17(8):e0271667. doi: 10.1371/journal.pone.0271667 (PMC9371331; doi:10.1371/journal.pone.0271667)

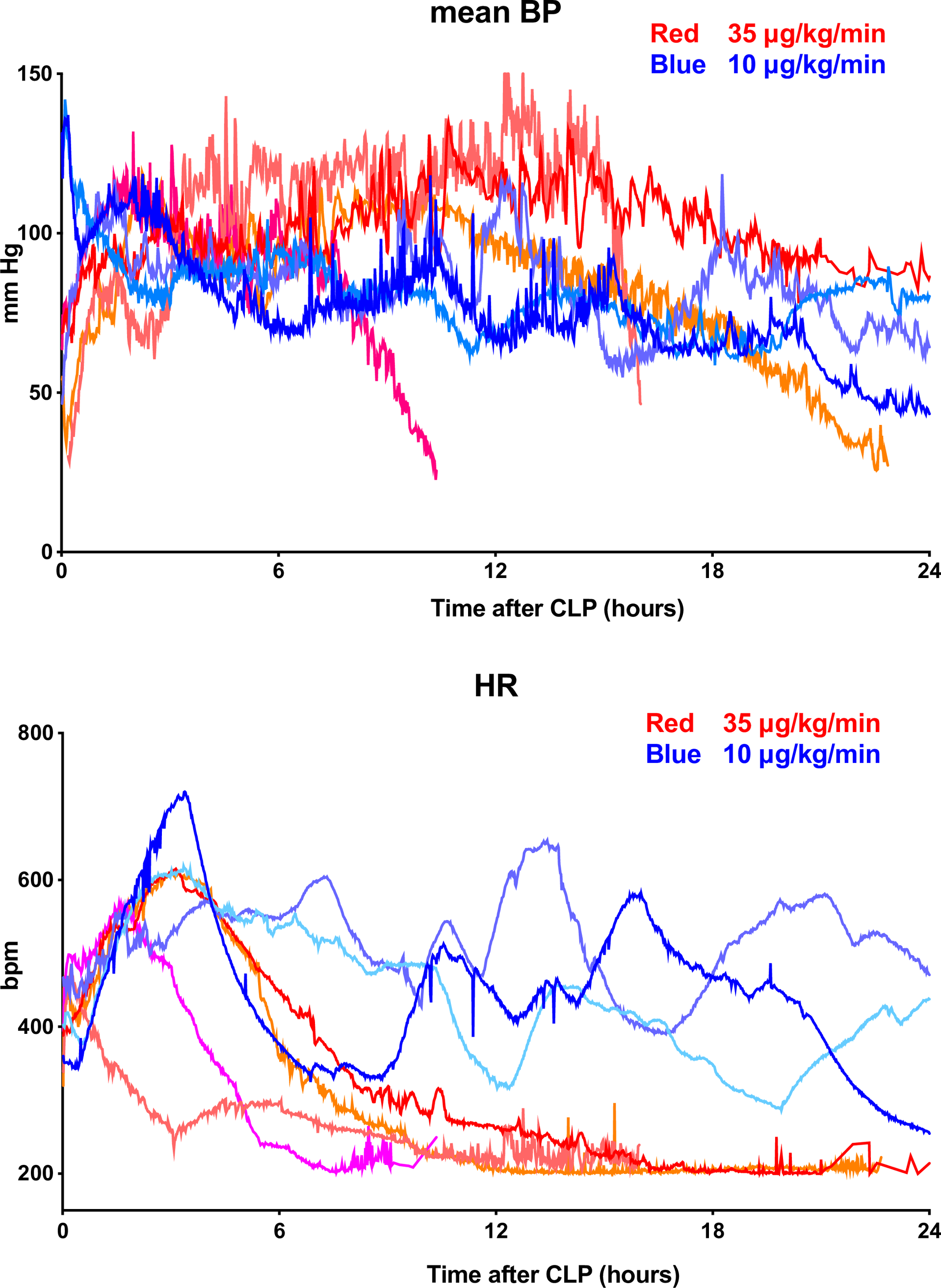

Supplement: S1 Fig — Different doses of norepinephrine were tested. Each line shows the data of each mice until 24 hours after cecal ligation puncture (CLP): mice treated with 35 ug/kg/min norepinephrine (NE) (red, light red, orange and purple; n = 4), and mice treated with 10 ug/kg/min NE (blue, light blue and pale blue; n = 3). A: mean arterial blood pressure (mean BP), B: heart rate (HR), times are synchronized by time after CLP surgery. (TIF) [file pone.0271667.s001.tif]

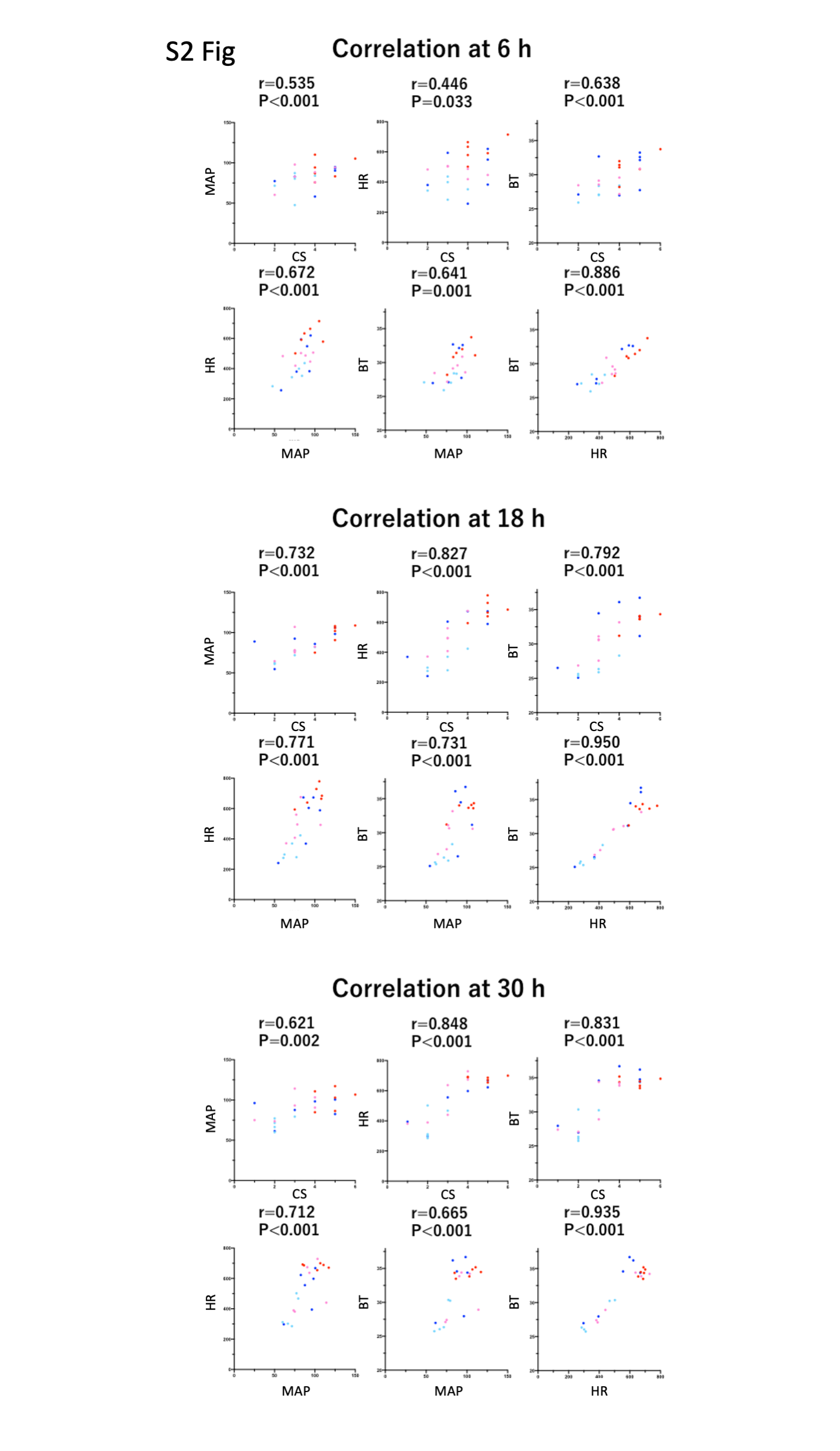

Supplement: S2 Fig — Each color indicates groups: survivors without norepinephrine (NE) treatment (dark blue; n = 7), mice that died within 7 days, without NE treatment (light blue; n = 5), survivors with NE treatment (dark red; n = 6), and mice that died within 7 days, with NE treatment (light red; n = 6). Clinical score ranged from 0 (no activity after stimuli) to 6 (active as normal). CS: clinical score, MAP: mean arterial blood pressure, HR: heart rate, BT: body temperature. Spearman’s correlation is used. (TIF) [file pone.0271667.s002.tif]

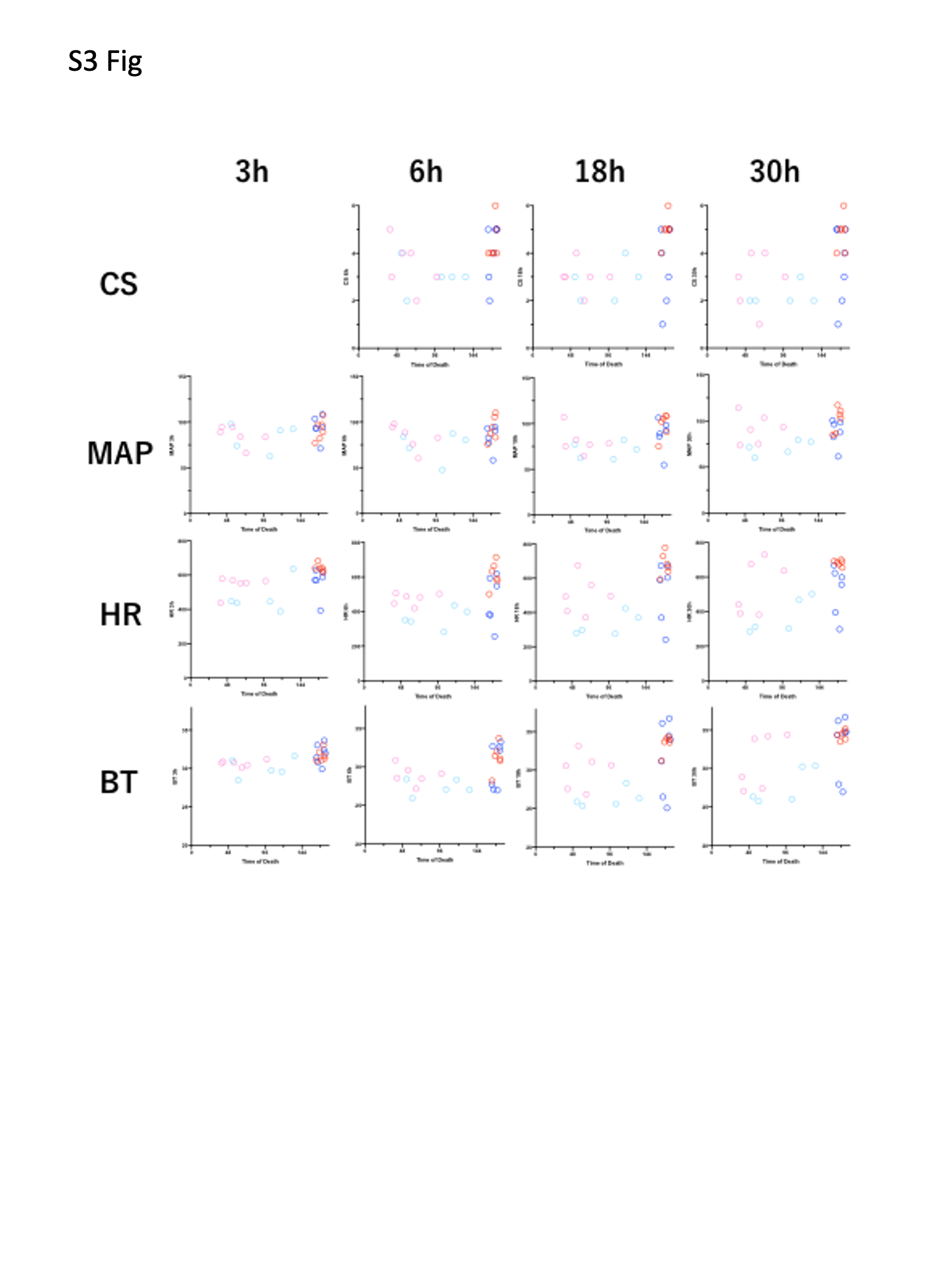

Supplement: S3 Fig — Each color indicates groups: survivors without norepinephrine (NE) treatment (dark blue; n = 7), mice that died within 7 days, without NE treatment (light blue; n = 5), survivors with NE treatment (dark red; n = 6), and mice that died within 7 days, with NE treatment (light red; n = 6). Clinical score was scored from 0 (no activity after stimuli) to 6 (active as normal). CS: clinical score, MAP: mean arterial blood pressure, HR: heart rate, BT: body temperature. (TIF) [file pone.0271667.s003.tif]

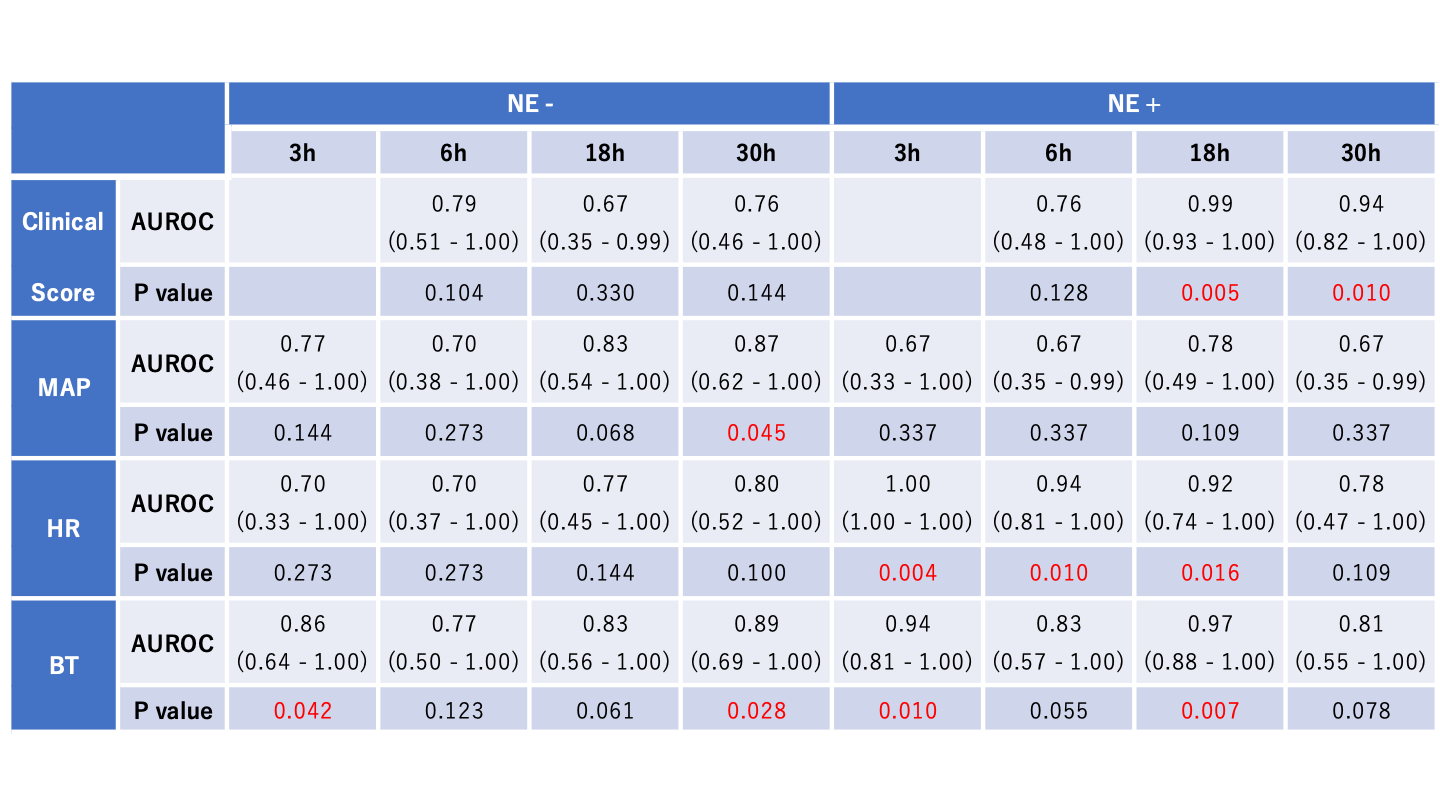

Supplement: S1 Table — (TIF) [file pone.0271667.s004.tif]

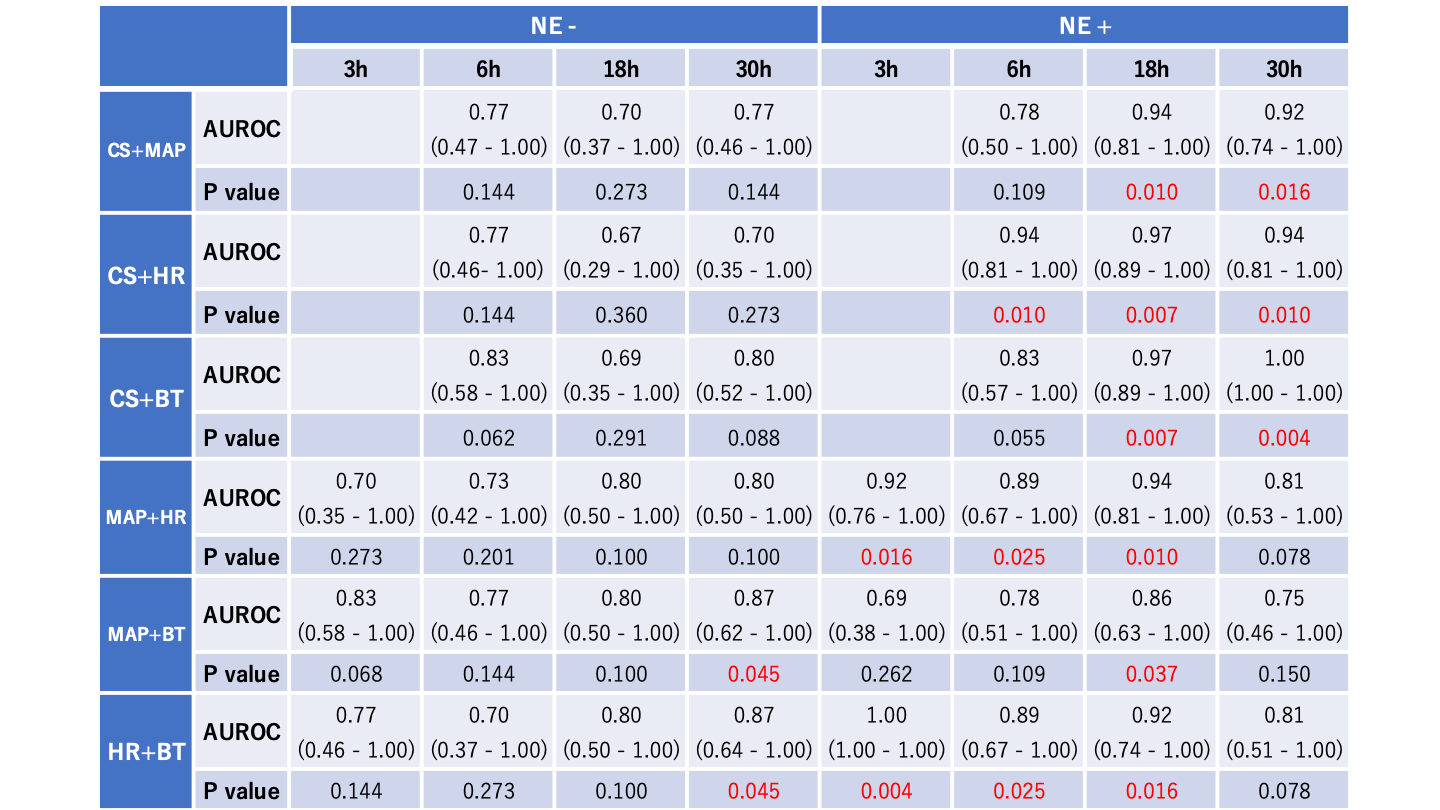

Supplement: S2 Table — To weight each parameter approximately equally, we used CS divided by 6, MAP divided by 150, HR divided by 800, and BT divided by 38 for analysis. (TIF) [file pone.0271667.s005.tif]

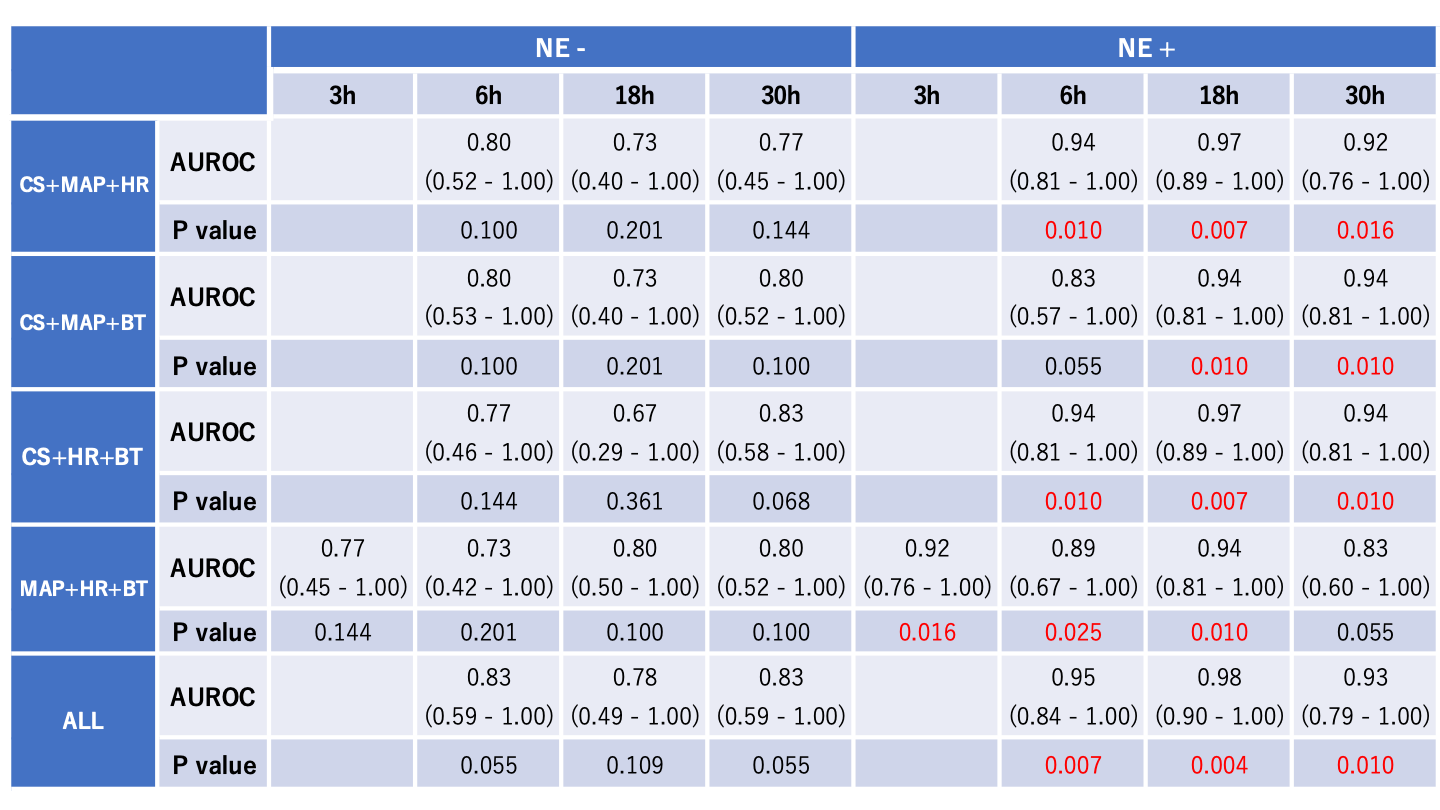

Supplement: S3 Table — To weight each parameter approximately equally, we used CS divided by 6, MAP divided by 150, HR divided by 800, and BT divided by 38 for analysis. (TIF) [file pone.0271667.s006.tif]
